# Scalable and straightforward synthesis of electrocatalysts for green hydrogen economy via high-current-density alkaline water splitting

**DOI:** 10.1093/nsr/nwaf417

**Published:** 2025-09-27

**Authors:** Zichen Xu, Yu Liang, Shisheng Yuan, Xiao Wang, Yun Zhao, Zhigang Shao, Zhong-Shuai Wu

**Affiliations:** State Key Laboratory of Catalysis, Dalian Institute of Chemical Physics, Chinese Academy of Sciences, Dalian 116023, China; State Key Laboratory of Catalysis, Dalian Institute of Chemical Physics, Chinese Academy of Sciences, Dalian 116023, China; Key Laboratory on Resources Chemicals and Materials of Ministry of Education of China, Shenyang University of Chemical Technology, Shenyang 110142, China; State Key Laboratory of Catalysis, Dalian Institute of Chemical Physics, Chinese Academy of Sciences, Dalian 116023, China; School of Materials Science and Engineering, Shenyang University of Chemical Technology, Shenyang 110142, China; State Key Laboratory of Catalysis, Dalian Institute of Chemical Physics, Chinese Academy of Sciences, Dalian 116023, China; Liaoning Binhai Laboratory, Dalian 116023, China; Fuel Cell System and Engineering Laboratory, Key Laboratory of Fuel Cell & Hybrid Power Sources, Dalian Institute of Chemical Physics, Chinese Academy of Sciences, Dalian 116023, China; Fuel Cell System and Engineering Laboratory, Key Laboratory of Fuel Cell & Hybrid Power Sources, Dalian Institute of Chemical Physics, Chinese Academy of Sciences, Dalian 116023, China; State Key Laboratory of Catalysis, Dalian Institute of Chemical Physics, Chinese Academy of Sciences, Dalian 116023, China; Liaoning Binhai Laboratory, Dalian 116023, China

**Keywords:** water electrolysis, hydrogen production, straightforward synthetic approach, scalable electrocatalyst, high current density

## Abstract

Water electrolysis for high-purity hydrogen production under alkaline conditions is essential for achieving hydrogen energy economy. Developing straightforward synthetic strategies for fabricating high-performance scalable electrocatalysts is pivotal for efficiency enhancement in water electrolysis at industrially relevant current densities (≥500 mA cm^−2^) and enabling cost-effective continuous electrocatalyst production. Herein, we summarize the recent advancements in scalable electrocatalyst construction for high-current-density water electrolysis. First, brief descriptions of various straightforward synthetic approaches are introduced, including hydrothermal and solvothermal synthesis, electrodeposition, corrosion engineering, Joule-heating and combustion, owing to their advantages of operational simplicity, effective modification, universality and scalability. Next, the recent advancements in developing scalable electrocatalysts using these approaches for high-current-density water electrolysis are overviewed and discussed. Lastly, current key challenges and potential future directions of these straightforward synthetic approaches are proposed. This review aims to offer new insights into designing and synthesizing innovative scalable electrocatalysts for industrial-scale hydrogen production.

## Teaser

This review article provides an overview of recent research advancements in the straightforward synthetic strategies for scalable electrocatalysts for high-current-density water electrolysis under alkaline conditions.

## INTRODUCTION

Overreliance on fossil fuels has caused severe energy, ecological and environmental crises [1,2]. The global push for carbon neutrality is driving the growing focus on employing clean, efficient and sustainable energy carriers to alleviate pressing issues [3,4]. Hydrogen (H_2_), a carbon-free energy, is viewed as an ideal substitute to fossil fuels owing to its abundant availability, widespread sources, high energy density and environmental friendliness [5–8]. However, the conventional H_2_ production technologies, such as steam reforming and coal gasification, are linked to considerable environmental impacts [9,10]. Given this circumstance, water electrolysis is gaining momentum as an effective route for sustainable H_2_ production [11,12].

In industrial-scale green H_2_ production, low-temperature (<100°C) water electrolysis operates via proton exchange membrane water electrolysis (PEMWE), anion exchange membrane water electrolysis (AEMWE) and alkaline water electrolysis (AWE) at elevated current densities of 0.5–1.6 A cm^−2^ under cell voltages of 1.8–2.4 V [13]. Although PEMWE has large current densities, high production rates and compact systems, it requires the use of scarce sources for fabricating catalysts (e.g. Pt, Ir and Ru) and components (e.g. Ti) owing to the severe corrosive acidic condition, which inevitably significantly increases H_2_ production costs and hinders large-scale implementation [14,15]. Compared to PEMWE, AEMWE and AWE enable utilization of non-precious metal-based materials, thereby reducing the catalyst and component costs for more economical large-scale H_2_ production [16–19]. Nevertheless, the further widespread implementation of AEMWE and AWE is severely limited by the sluggish kinetics and inefficiency of the cathodic hydrogen evolution reaction (HER) and anodic oxygen evolution reaction (OER) [20–22]. To date, various electrocatalysts have been reported, exhibiting low overpotentials and rapid reaction kinetics. However, most of them perform at ≤200 mA cm^−2^, not satisfying the typical industrial requirements (usually ≥500 mA cm^−2^) [23–25]. Also, the electrocatalysts undergo more rigorous durability tests under industrial operating conditions [15,26]. Moreover, it is projected that the future global H_2_ demand will greatly surpass the current production capacity with the rapid development of society [27]. Therefore, there has been growing attention on developing high-performance electrocatalysts for high-current-density AEMWE and AWE.

Along with achieving outstanding performance at industrial-level current densities, the electrocatalyst preparation procedure is equally important. In general, electrocatalysts can be effectively tuned for better performance by combining different strategies via a multi-step preparation process, and some of the related research findings show that the resulted electrocatalysts can attain a current density of at least 1000 mA cm^−2^ [28–34]. Even so, such preparation involves complex and cumbersome reaction steps, and time-consuming post-treatment processes, which result in a substantial waste of time and energy, as well as poor reproducibility [35–38]. This will significantly increase fabrication costs, reduce efficiency and jeopardize environmental sustainability. Aside from this, scalability should also be given consideration. Currently, the vast majority of studies focus exclusively on electrocatalytic performance while largely neglecting scalability; most of the electrocatalysts are prepared in at the scale of milligrams (≤100 mg) or several square centimeters (≤5 cm^2^), creating a substantial gap between the pilot-scale applications (decimeters or grams) and industrial requirements (square meters or kilograms) [39–42]. Considering these concerns, exploring straightforward synthetic approaches is essential, as they offer cost-effectiveness, fast synthesis and high efficiency, contributing to the development of water electrolysis under high current densities.

**Figure 1.**
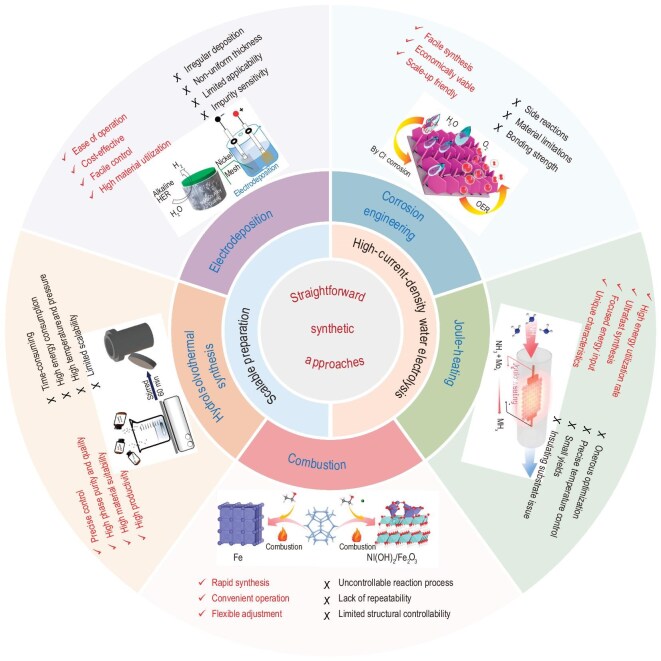
Straightforward synthetic approaches for preparing high-current-density scalable electrocatalysts. Reprinted with permission from Mao *et al.* [43]. Copyright 2023, American Chemical Society. Reprinted with permission from Abdulhamid *et al.* [44]. Copyright 2023, Springer Nature. Reprinted with permission from Hao *et al.* [45]. Copyright 2019, American Chemical Society. Reprinted with permission from Shen *et al.* [46]. Copyright 2024, Springer Nature. Reprinted with permission from Yu *et al.* [47]. Copyright 2023, American Chemical Society.

Recent studies have demonstrated the great promise of straightforward synthetic approaches, including electrodeposition, corrosion engineering, hydrothermal synthesis and solvothermal synthesis etc., for the scalable production of high-performance electrocatalysts, particularly for water electrolysis at industrial-level current densities. To this end, the primary focus of this review is to present an overview of recent advancements in design and synthesis of scalable electrocatalysts for high-current-density water electrolysis via various straightforward synthetic approaches (Fig. 1). The review first outlines the diverse straightforward synthetic approaches, mainly based on the basic concept, implementation and determinants. Subsequently, the significant development of these approaches for scalable anodic and/or cathodic electrocatalyst preparation are presented. Finally, the current existing issues and potential future avenues in the straightforward and scalable synthesis of electrocatalysts for industrial-level water electrolysis are presented.

## OVERVIEW OF STRAIGHTFORWARD SYNTHETIC APPROACHES

### Hydro/solvothermal synthesis

Hydrothermal and solvothermal synthesis are important synthetic approaches in materials science and chemistry [48]. Since the 1800s, innovative hydrothermal and solvothermal synthesis processes have been developed for new material synthesis. In modern chemistry, hydrothermal and solvothermal synthesis involve chemical reactions carried out in custom-made sealed reaction vessels, designed to simulate high-temperature and high-pressure environments using heated aqueous or organic solutions [49,50]. Typically, water is the solvent for hydrothermal synthesis, whereas organic solvents or a mixture of organic solvents and water are the solvents for solvothermal synthesis.

The properties of the target materials, including crystalline structure, morphologies and particle sizes etc., can be affected by temperature, reaction time, solvent compositions and reductant effects etc. [51]: (i) temperature and reaction time. The characteristics of solvents, including the viscosity, mobility and dielectric constant etc., can be changed with temperature, leading to the formation of different outcomes. Varying reaction time is associated with distinct formation stages, where morphology evolution often occurs as the reaction time increases [52]; (ii) solvent. The compositions, polarities, ratios and volumes of mixed solvents can expand the possibilities for material synthesis, as well as morphology control; and (iii) reductant. The use of reductants also offers the possibility for controlling the shape and morphology via tuning the reduction kinetics by adjusting pH and reductant concentration [53].

So, hydrothermal and solvothermal synthesis enable high phase purity, fast reaction rates, high productivity and environmental sustainability, establishing them as predominant synthetic approaches [54]. Nonetheless, several issues still remain to be addressed during the electrocatalyst preparation, including the time-consuming and energy-intensive nature and limited scalability.

### Electrodeposition

Electrodeposition, originating in the 19th century, is an electrochemical technique involving the cathodic reduction reaction and the anodic oxidation reaction driven by applied electric energy, and has garnered significant attention [55]. It started as an effective way to analyze the electrode performance, and has progressively developed into a material synthetic technology [56,57]. Owing to its excellent technical flexibility, the structure and composition of the resulting materials are optimized via tuning electrolytes and technical parameters. Taking self-supporting electrocatalysts as an example, the active species can be uniformly electrodeposited on the skeleton of the support within a short period, realizing fast electron transfer and improved stability of electrocatalytic reactions [58,59]. In addition, synthesizing amorphous materials is also quite simple via electrodeposition, in which as-obtained amorphous materials possess rich defects and unique electronic structures that enhance the electrocatalytic performance [60,61].

The microstructures and compositions of the resulting materials can be flexible, tailored by regulating different parameters as follows: (i) the electricity input mode. It is directly related to the nucleation. The most common method is the potentiostatic and galvanostatic input with continuously maintained voltage or current [62]. The reaction rate, coating adhesion and system stability are relevant to the set voltage or current, thereby affecting the morphology and composition, ultimately influencing the activity. Through the current response to the voltages to carry out the reciprocating oxidation/reduction process, cyclic voltammetry (CV) effective deposition can also be realized, especially for single-atom catalysts (SACs) [63,64]. Apart from the aforementioned methods, pulse current or voltage input is also a feasible way through exerting periodical current or voltage under certain frequency [65]. By adjusting the frequency and current or voltage, the crystallization behavior of the nucleus can be controlled, which in turn affects the performance [66]; (ii) the electrolyte. Tuning the parameters of the electrolytes will directly affect the deposition process [67]. The solvent and solute are the two primary components of the electrolyte. For the general requirements, water is the most commonly used solvent. Ionic liquids and deep eutectic solvents also serve as suitable solvents [68,69]. As for the solute, it can be hydrolyzed into anions and cations to serve as reactants to participate in deposition. The adjustment of the concentration of solute will affect ion diffusion behavior, and thereby impact the properties and performance of the resulting materials [70,71]. Apart from solvent and solute, regulating temperature, pH and time is equally important. The reaction thermodynamics controlled by temperature influence the reaction rate and efficiency [72]. Additionally, electrolyte pH determines which reaction types occur, which are directly related to the target materials [73]. When it comes to the length of time, it has an impact on the electrodeposition thickness and structure size, where optimal deposition time will induce the ideal performance [74]; and (iii) the substrate and template. The utilization of multi-dimensional substrates, including fluorine-doped SnO_2_, carbon cloth and paper, and metal foam (nickel, cobalt, iron, and copper etc.), not only provides high electrical conductivity but also offers abundant pore structure, contributing to high specific surface areas [75,76]. In terms of the template, its adoption also contributes to increasing specific surface area and enriching catalytic active sites [77]. Moreover, the deposited materials exhibit synergetic effects with the substrates or templates that can optimize the reaction kinetics for better performance [78].

To sum up, the above-mentioned parameters are interconnected and collectively influence electrodeposition. With suitable adjustments, unforeseen performance may be demonstrated in scalable electrocatalysts. However, it should also be noted that electrodeposition is only applicable to limited materials, and irregular deposition and non-uniform thickness issues may emerge, especially for large-scale preparation.

### Corrosion engineering

As a naturally occurring phenomenon, corrosion is highly undesirable in industrial production [79]. However, corrosion layers generated via the spontaneous oxidation–reduction can serve as effective active materials for electrochemical reactions. For example, the most common rust is primarily composed of the FeOOH phase, which has been demonstrated as an active material in the OER [80–82]. Thus, it can be envisaged that corrosion engineering holds great promise for fabricating electrocatalysts.

During the corrosion process, the metal ions are released from the metal substrates upon the introduction of strong corrodents (i.e. O_2_, H_2_O_2_, Fe^3+^ and S_2_O_8_^2−^), and corrosion layers are formed through the coordination with hydroxyl groups or other electronegative radicals [83,84]. The corrosion-engineered materials have a rough surface and high hydrophilicity, allowing high active-site utilization and rapid charge transfer to accelerate electrocatalytic reactions [85–87]. Generally, the corrosion processes can be affected by various factors as follows: (i) the solution composition and concentration. The corrosion layers are usually formed with the participation of cations and anions, where metal cations are responsible for generating target corrosion products, and anions maintain charge balance, both of which influence the compositions and intrinsic structures. Thus, the solution compositions need to be taken into account. Also, the solution concentration will affect the structure of the corrosion layer and corrosion processes [88,89]; (ii) the diffusion rate. Briefly, the corrosion involves diffusion of the ions and O_2_. Thus, proper stirring will enhance the reaction rate, reduce corrosion time and facilitate uniform species distribution; (iii) the ambient atmosphere. Since O_2_ can act as a corrodent, maintaining sufficient O_2_ is crucial for electrocatalyst synthesis, and sustained stirring contributes to O_2_ supplementation [90]; and (iv) the reaction time and temperature. Longer reaction time and higher temperature can favor the corrosion process [91]. For instance, with increasing reaction time, the morphology, structure and crystallinity of the corrosion layer will evolve [92].

All in all, an optimal thickness of the corrosion layer can be obtained through the rational adjustment of various factors, leading to the ideal electrocatalytic performance. However, similar to electrodeposition, corrosion engineering also has limited material applicability. Besides this, it also suffers from occurrence of side reactions and bonding strength issues.

### Other straightforward synthetic approaches

Beyond the approaches outlined above, some novel techniques have also emerged in recent years for the straightforward and efficient scalable synthesis of electrocatalysts. For instance, through Joule heating, temperatures can be raised to 3000 K within a few milliseconds, in contrast to the conventional thermal processing that takes hours or even days [93]. Meanwhile, the energy input is more precise and targeted, contributing to higher energy efficiency, imparting unique properties to the resulting materials [94,95]. As a natural phenomenon, combustion is also a feasible approach for electrocatalyst preparation owing to its ability to rapidly generate sufficient thermal energy [96]. For the self-supporting electrocatalysts, firm contact will be established between the electrocatalyst and substrate, guaranteeing excellent electrocatalytic activity and long-term stability [97].

In short, Joule heating and combustion provide more options for straightforward synthesis of scalable electrocatalysts, while also improving preparation efficiency. Notwithstanding this, they also encounter several challenges, such as onerous trial-and-error optimization and precise control for Joule heating, and limited repeatability and controllability for combustion.

To clearly compare the various synthetic approaches mentioned above, their advantages, drawbacks and potential industrial relevance (e.g. fabrication cost, efficiency, scalability, material suitability and environmental sustainability) are summarized in Table 1.

**Table 1.** Comparison of different straightforward synthetic approaches.

			Potential industrial relevance				
Synthetic approach	Advantage	Disadvantage	Fabrication costs	Efficiency	Scalability	Material suitability	Environmental sustainability
Hydro/solvothermal synthesis	Precise control over size, shape, and morphology High phase purity and quality High productivity High material suitability	Time-consuming High energy consumption High temperature and pressure requirements Limited scalability	High	Low	Low	High	Low
Electrodeposition	Ease of operation Cost-effective Straightforward control over structure and thickness High material utilization	Irregular deposition Non-uniform thickness Limited material applicability Impurity sensitivity	Medium	High	High	Medium	Medium
Corrosion engineering	Straightforward synthesis route Economically viable Scale-up friendly	Occurrence of side reactions Material limitations Bonding strength issues	Low	Medium	High	Low	Medium
Joule heating	High energy utilization rate Ultrafast synthesis Precise and targeted energy input Materials with unique characteristics	Onerous trial-and-error optimization Precise temperature measurement Limited reactor size (small yields) Challenges in non-conductive substrates	High	High	Medium	Medium	Medium
Combustion	Rapid synthesis Convenient operation Flexible adjustment	Uncontrollable reaction process Lack of repeatability Limited structural controllability	Low	High	High	Low	Medium

## STRAIGHTFORWARD SYNTHETIC APPROACHES FOR INDUSTRIAL-LEVEL WATER ELECTROLYSIS

So far, various straightforward synthetic approaches have been developed for large-scale fabrication of electrocatalysts for high-current-density water electrolysis, thereby targeting industrial applications. Thus, the recent advancements of these approaches are presented in detail here.

### Hydrothermal or solvothermal synthesis

**Figure 2.**
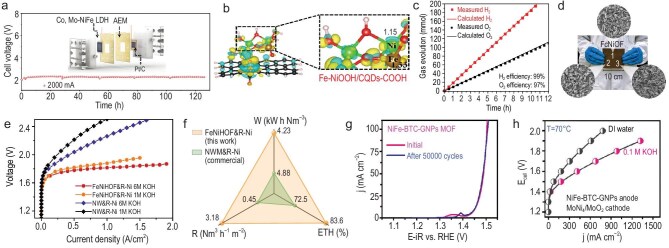
Hydro/solvothermal synthesis of scalable electrocatalysts for high-current-density water electrolysis. (a) Chronopotentiometry curve of Co, Mo-NiFe LDH-based AEMWE device at 2000 mA. Reprinted with permission from Zhao *et al.* [98]. Copyright 2023, Wiley‐VCH. (b) Charge distributions in Fe-NiOOH/CQD-COOH. (c) Measured and calculated O_2_ and H_2_ evolution. (b and c) Reprinted with permission from Pan *et al.* [99]. Copyright 2022, Wiley‐VCH. (d) Photograph of 90 cm^2^ FcNiOF and SEM images at different positions. (e) LSV curves for water electrolysis. (f) Energy efficiency contrast. (d–f) Reprinted with permission from Chen *et al.* [100]. Copyright 2024, Springer Nature. (g) LSV curves of NiFe-BTC-GNPs before and after 50 000 CV. (h) LSV curves for water electrolysis. (g and h) Reprinted with permission from Thangavel *et al.* [101] Copyright 2020, Royal Society of Chemistry. ETH, energy efficiency; E-iR, potential with iR-compensation; j, current density; R, H_2_ yield rate; RHE, reversible hydrogen electrode; W, power consumption.

In terms of hydrothermal synthesis, Zhao *et al.* verified via density functional theory (DFT) that metal cation vacancy incorporation and metal doping tuned the *d*-band centers, leading to optimized binding energies of intermediates, a benefit for achieving low potentials [98]. In view of the theoretical findings, the Co, Mo co-doped NiFe layered double hydroxide (Co, Mo-NiFe LDH) was hydrothermally synthesized directly. During the subsequent electrochemical testing, Co, Mo-NiFe LDH underwent *in situ* reconstruction into Co-doped NiFe oxyhydroxide with metal site vacancy (Co, V_M_-NiFe OOH) due to Mo leaching, which served as the real active electrocatalyst. In 1.0 M KOH, Co, Mo-NiFe LDH achieved high electrocatalytic activity (272 mV @ 200 mA cm^−2^) and rapid kinetics (43 mV dec^−1^). Furthermore, AEMWE and AWE performance were also evaluated. The 4 cm^2^ AEMWE device with Co, Mo-NiFe LDH and Pt/C showed a cell voltage of 1.94 V at 2000 mA, surpassing RuO_2_||Pt/C, and demonstrated outstanding durability for 130 h (Fig. 2a). In the case of AWE, Co, Mo-NiFe LDH with a diameter of 5 cm was used. The assembled AWE electrolyzer (Co, Mo-NiFe LDH||Raney Ni) only needed 1.91 V at 400 mA cm^−2^, and almost no performance loss in 100 h. Likewise, Pan *et al.* reported Ni/Fe-doped carbon quantum dots (NiFe-CQDs) via a one-step hydrothermal approach [99]. Similarly, NiFe-CQDs were transformed into active Fe-NiOOH/CQDs during the electrochemical activation. Using Ni foil as the substrate, 450 mV was reached at 1000 mA cm^−2^. The DFT calculations revealed that the strong metal–substrate interaction between CQDs and Fe-NiOOH induced the regulated electronic environment and regulated energy barriers in the OER (Fig. 2b). Additionally, contact angle measurements confirmed its high hydrophilicity and aerophobicity, which facilitated electrolyte penetration and bubble dissipation. When coupled with Pt/C, NiFe-CQDs||Pt/C offered 1.85 V at 100 mA cm^−2^ and high Faradaic efficiency (∼100%) (Fig. 2c). Significantly, the straightforward synthesis allowed for scalable production of NiFe-CQDs.

Relying on the solvothermal approach, ferrocene–nickel organic framework (FcNiOF) nanosheet arrays were fabricated on nickel foam (NF) by Xu’s group [100]. Owing to the ease of the synthetic process, FcNiOF preparation was successfully scaled up, with an area of 90 cm^2^ (Fig. 2d). The subsequent CV activation induced FcNiOF transformation into ferro–nickel hydroxide organic framework (FeNiHOF), displaying 340 mV @ 2000 mA cm^−2^. When FeNiHOF was further combined with Raney Ni for AWE, 1.87 and 1.81 V were achieved at 1000 mA cm^−2^ in 1.0 and 6.0 M KOH, respectively (Fig. 2e). It also realized low power consumption of ∼4.23 kW h Nm^−3^ and high energy efficiency of ∼83.6%, highlighting its actual application prospects (Fig. 2f). Also, for metal–organic frameworks (MOFs), Thangavel and co-workers realized the scalable synthesis of graphene nanoplatelet (GNP)-supported bimetallic NiFe 1,3,5-benzenetricarboxylate MOFs (NiFe-BTC-GNPs MOF) [101]. It showed 220 mV at 10 mA cm^−2^ (Fig. 2g), which was further lowered to 180 mV when loaded on NF. In the further tests on AEMWE using anodic NiFe-BTC-GNPs MOF and cathodic MoNi_4_/MoO_2_, 1.85 V was realized at 540 mA cm^−2^ [deionized (DI) H_2_O, 70°C] and 1150 mA cm^−2^ (0.1 M KOH, 70°C), respectively (Fig. 2h). During the stability measurement, it verified high stability retention of 95.8% and a low degradation rate of 0.27 mA h^−1^. Furthermore, the Faradaic efficiency for O_2_ generation could be as high as 98.6%.

As shown above, straightforward hydrothermal and solvothermal synthesis, being among the most widely used approaches for material synthesis, significantly drive the fast development of water electrolysis, particularly for scalable electrocatalyst preparation. However, since the synthesis process usually takes several to tens of hours, it is not favorable for continuous preparation.

### Electrodeposition

**Figure 3.**
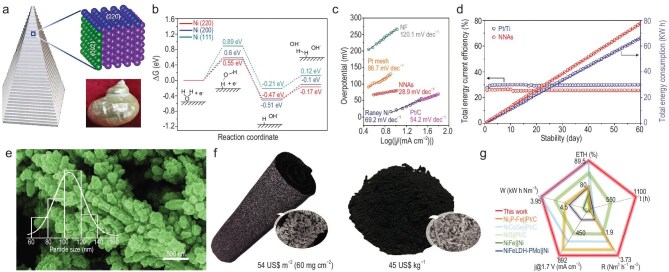
Electrodeposition of scalable electrocatalysts for high-current-density water electrolysis. (a) Scheme for the NNAs’ step surface facet composition, and photograph of a spiral conch. (b) Calculated HER adsorption free energy. (c) HER Tafel plots. (d) Relationship between total energy consumption and total energy current efficiency. (a–d) Reprinted with permission from Lei *et al.* [103]. Copyright 2023, Wiley‐VCH. (e) SEM image of ND-Ni. Inset: the grain size distribution. (f) ND-Ni on NF and in powder of ND-Ni, and corresponding prices. Insets: SEM images. (g) Performance comparison. (e–g) Reprinted with permission from Zhu *et al.* [104]. Copyright 2023, Wiley‐VCH. ETH, electricity-to-hydrogen energy conversion efficiency; j, current density; R, hydrogen production rate; W, ydrogen yield.

For pure metals, the surface low-coordinated atoms enhance the electrocatalytic performance [102]. In view of this, Yang’s group adopted a non-equilibrium screw dislocation strategy to electrodeposit pure nickel nanopyramid arrays (NNAs) on NF, aiming to expose surface low-coordinated atoms to enhance HER performance [103]. Based on crystallographic analysis, it is deduced that horizontal (220) and vertical (002) facets exist in NNAs, analogous to the biological nacreous layer, which could contribute to exceptional durability (Fig. 3a). It is further revealed by DFT calculations that Ni (220) had optimal Gibbs free energy of hydrogen adsorption, which could exhibit excellent reaction kinetics (Fig. 3b). Consequently, NNAs presented outstanding electrocatalytic HER activity (469 mV @ 5000 mA cm^−2^), low Tafel slope (28.9 mV dec^−1^) and prolonged stability (1000 mA cm^−2^ over 7000 h) in 1.0 M KOH (Fig. 3c). For universal applicability, NNAs were also electrodeposited on various metal substrates. As for large-scale electrode fabrication, NNAs could be mass produced by a pilot-scale setup, yielding a size of 20 × 100 cm on NF. Furthermore, when 161 cm^2^ NNAs were applied as the cathode in the HFH-300 hydrogen generator [anode: commercial stainless steel wire mesh (SSM)], it worked steadily at 250 mA cm^−2^ for 60 days (10 wt% KOH, 55°C−60°C) with negligible change in the HER activity (Fig. 3d). Similarly, regarding pure nickel, Xu’s group developed an orderly nanodendritic nickel (ND-Ni) on NF via electrodeposition [104]. Small nanograins with average sizes of 106 nm were linked together to form the dendritic structure of ND-Ni, and the lattice blending was between the nanograin interfaces that supported the stable electrocatalytic operation (Fig. 3e). Also, ND-Ni exhibited large porosity, high electronic conductivity and superhydrophilicity, which collectively improved HER kinetics and efficiency. Thanks to the aforementioned structural characteristics, ND-Ni exhibited high electrocatalytic activity and superior durability. In addition, whether in powder or film, ND-Ni could be easily scaled up at a lower cost (∼$45 kg^−1^ for powder, ∼$54 m^−2^ for film) compared to the commercial 10 wt% Pt/C (∼$31 000 kg^−1^) and Raney Ni (∼$50 kg^−1^) (Fig. 3f). Furthermore, when ∼20 cm^2^ ND-Ni was coupled with NiFeOH (NiFeOH||ND-Ni) for AWE, an ultralow cell voltage of 1.79 V @ 1000 mA cm^−2^ was realized in 1.0 M KOH at ambient temperature, which could be reduced to 1.71 V @ 1000 mA cm^−2^ in 6.0 M KOH at ∼80°C. The pair worked steadily for at least 1100 h with degradation of ∼0.08 mV h^−1^ (6.0 M KOH, ∼80°C). Significantly, the low power consumption (3.95 kW h Nm^−3^) and high energy conversion efficiency (89.5%) of NiFeOH||ND-Ni highlighted its great application value (Fig. 3g).

**Figure 4.**
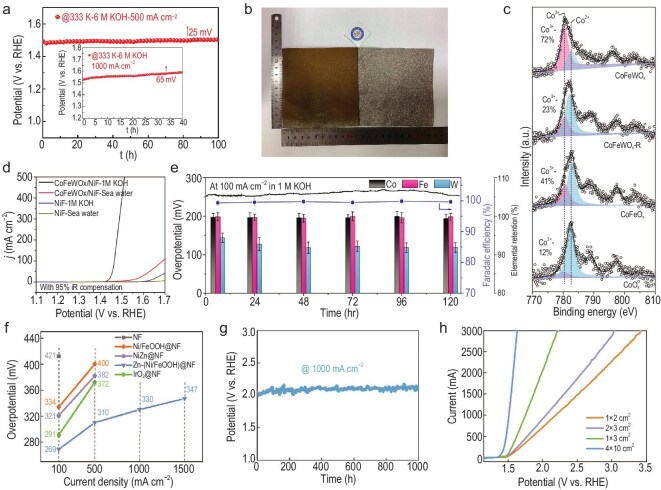
Electrodeposition of scalable electrocatalysts for high-current-density water electrolysis. (a) Durability at 500 mA cm^−2^. Inset: durability at 1000 mA cm^−2^. (b) Large-area FeCoNiMnOOH/NF. (a and b) Reprinted with permission from He *et al.* [108]. Copyright 2022, Elsevier. (c) High-resolution Co XPS spectra. (d) OER LSV curves. (e) Stability at 100 mA cm^–2^ in 1.0 M KOH, and the corresponding Co, Fe and W retention. (c–e) Reprinted with permission from Chen *et al.* [109]. Copyright 2020, Wiley‐VCH. (f) Overpotentials at 100, 500, 1000 and 1500 mA cm^−2^. (g) Durability at 1000 mA cm^−2^. (h) OER LSV curves of 2, 3, 6 and 40 cm^2^ Zn-(Ni/FeOOH)@NF electrode. (f–h) Reprinted with permission from Zhang *et al.* [110]. Copyright 2022, Wiley‐VCH. RHE, reversible hydrogen electrode.

Representing a class of highly effective OER electrocatalysts, metal (oxy)hydroxides have been extensively reported [105–107]. In this regard, electrodeposition offers a rapid and straightforward pathway for the efficient synthesis of metal (oxy)hydroxides. For instance, He *et al.* applied electrodeposition for synthesis of various high-entropy (oxy)hydroxides on NF (FeCoNiMnOOH/NF) in just 20 min [108]. Among these, FeCoNiMnOOH/NF displayed the optimal OER activity (441 mV @ 1000 mA cm^−2^) and excellent durability in 1.0 M KOH (100 and 500 mA cm^−2^ @ 200 h) and 6.0 M KOH (500 mA cm^−2^ @ 100 h, 60°C) (Fig. 4a). Also, given the convenience and scalability of the synthetic route, FeCoNiMnOOH/NF with an area of ≥100 cm^2^ was fabricated (Fig. 4b). With a shorter electrodeposition time of 10 min, amorphous Co–Fe–W oxyhydroxides (CoFeWO_x_) were fabricated by Chen’s group, where the abundance of highly OER-active octahedrally coordinated Co^3+^ (Co_Oh_^3+^) could be effectively tuned [109]. As a result, the abundance of Co_Oh_^3+^ in CoFeWO_x_ was identified as 72% (Fig. 4c), which enabled excellent OER activity (Fig. 4d). DFT calculations demonstrated that the synergy between Fe- and W-regulated CoOh^3+^ fine-tuned interactions and reaction intermediates, thereby boosting activity. When CoFeWO_x_ and NiMoO_4_ were used to assemble a water electrolyzer, it attained outstanding durability in 1.0 M KOH (100 mA cm^−2^ @ 120 h) (Fig. 4e) and 10.0 M KOH (500 mA cm^−2^ @ 48 h, 80°C). Additionally, CoFeWO_x_ could also be scalably synthesized with an area of 60 cm^2^. With the electrodeposition time further shortened to 500 s, Zn-doped NiOOH-FeOOH was designed on NF [Zn-(Ni/FeOOH)@NF] by Zhang *et al.* [110]. Scanning electron microscopy (SEM) confirmed the thickness of the deposition layer on NF, ensuring efficient bubble nucleation and dissipation, resulting in the superhydrophilicity and superaerophobicity of Zn-(Ni/FeOOH). Therefore, Zn-(Ni/FeOOH)@NF reached excellent OER activity and stability at 1000 mA cm^−2^ (Fig. 4f and g). Moreover, it was also fabricated with areas of 2, 3, 6 and 40 cm^2^, and the maximum current could reach 3000 mA at 405 mV (Fig. 4h). In brief, electrodeposition has emerged as an extensively adopted technique for scalable electrocatalyst fabrication. Beyond the above, numerous related studies have further highlighted its immense potential in water electrolysis [111,112].

### Corrosion engineering

**Figure 5.**
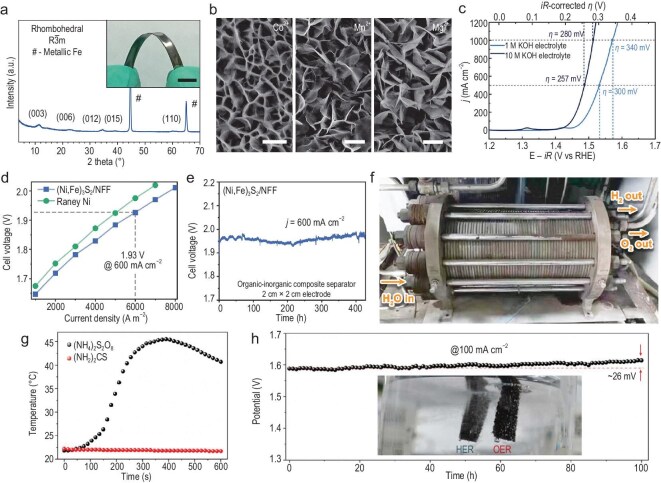
Corrosion engineering of scalable electrocatalysts for high-current-density water electrolysis. (a) XRD pattern of the electrocatalyst using Ni^2+^. Inset: The digital image (scale bar: 1 cm). (b) SEM images of the electrocatalyst-obtained Co^2+^, Mn^2+^ and Mg^2+^. Scale bars: 400 nm, 1 μm and 1 μm. (c) OER LSV curves. (a–c) Reprinted with permission from Liu *et al.* [83]. Copyright 2018, Springer Nature. (d) Polarization curves for alkaline electrolyzers. (e) Durability at 600 mA cm^−2^. (f) Photograph of an industrial alkaline electrolyzer with a diameter of 20 cm. (d–f) Reprinted with permission from Bai *et al.* [114]. Copyright 2024, Wiley‐VCH. (g) The trend of the solution temperature with increased time. (h) Stability at 100 mA cm^−2^. (g and h) Reprinted with permission from Chen *et al.* [84]. Copyright © 2023, Royal Society of Chemistry. E-iR, potential with iR-compensation; j, current density; RHE, reversible hydrogen electrode; η, overpotential.

For fabricating larger-sized electrocatalysts, hydro/solvothermal synthesis faces equipment size limitations, while electrodeposition suffers from uneven current distribution [113]. In this regard, corrosion engineering provides an economical, convenient and feasible approach. Representatively, Zou’s group immersed iron substrates into divalent metal cation solutions for large-scale synthesis of Fe-containing LDHs [83]. Considering the versatility of the process, various LDHs, including NiFe-LDH, CoFe-LDH, MnFe-LDH and MgFe-LDH with nanosheet structures, were vertically formed on iron plate surfaces (Fig. 5a and b). Taking NiFe-LDH as an example, it not only achieved large-scale synthesis (0.1 m × 1 m) with uniform nanosheet arrays but also demonstrated high activity (269 mV @ 10 mA cm^−2^) and durability (100 mA cm^−2^ @ 100 h) in 1.0 M KOH. When using iron foam (IF) to construct 3D-O_2_-Cat-1, the electrocatalytic activity could be further improved to 340 mV (1.0 M KOH) and 280 mV (10.0 M KOH) @ 1000 mA cm^−2^ (Fig. 5c). Remarkably, its long-term stability was further enhanced, achieving 5000 h (1.0 M KOH) and 1050 h (10.0 M KOH) @ 1000 mA cm^−2^, validating its exceptional electrocatalytic stability. To further target industrial water electrolysis, Zou’s group fabricated (Ni, Fe)_3_S_2_ on NiFe foam (NFF), termed (Ni, Fe)_3_S_2_/NFF, by immersing NFF into an Fe^3^⁺- and S_2_O_3_^2−^-containing solution [114]. Similarly, (Ni, Fe)_3_S_2_/NFF was also scaled up with a size of 1.0 m × 0.5 m with homogeneous distribution of (Ni, Fe)_3_S_2_ nanosheets. When it was assessed in 30% KOH, it offered 241 mV for OER and 168 mV for HER at 100 mA cm^−2^. Besides this, it also achieved high Faradaic efficiency of 96% and exhibited excellent durability for both OER and HER. Furthermore, 4 cm^2^ (Ni, Fe)_3_S_2_/NFF was employed in an alkaline electrolyzer; it afforded a low cell voltage of 1.93 V and high durability of 420 h at 600 mA cm^−2^ (Fig. 5d and e). Beyond that, (Ni, Fe)_3_S_2_/NFF with a diameter of 20 cm was also employed in an industrial alkaline electrolyzer (Fig. 5f), exhibiting ∼1.95 V at 300 mA cm^−2^.

Unlike the corrosion engineering proposed by Zou’s group, Chen *et al.* exploited the heat rapidly generated by employing an (NH_4_)_2_S_2_O_8_-, (NH_2_)_2_CS- and FeCl_3_-containing solution to corrode NFF to produce defect-rich Ni(Fe)OOH/Ni(Fe)S_x_ [84]. Its defects were demonstrated by high-resolution transmission electron microscopy (HRTEM), where fuzzy and disordered lattice fringes were presented, which was beneficial for modulating the electronic structures to boost electrocatalytic activity. Also, a large-sized electrode of 10 cm × 10 cm was fabricated, which maintains a consistent nanosheet array morphology. Further experimental results showed that its formation could be attributed to the rapid reaction between (NH_4_)_2_S_2_O_8_ and NFF, which generated significant heat (Fig. 5g), accelerating the hydrolysis of (NH_2_)_2_CS and releasing OH^−^ and S^2−^. The OER performance was determined to be 313 mV at 1000 mA cm^−2^. When further coupled with Mo_2_C/MoO_2_/MoNi_4_ for water electrolysis, the resulting pair only delivered 1.809 V at 1000 mA cm^−2^ and exhibited long-term stability at 100 mA cm^−2^ for 100 h in 1.0 M KOH (Fig. 5h).

**Figure 6.**
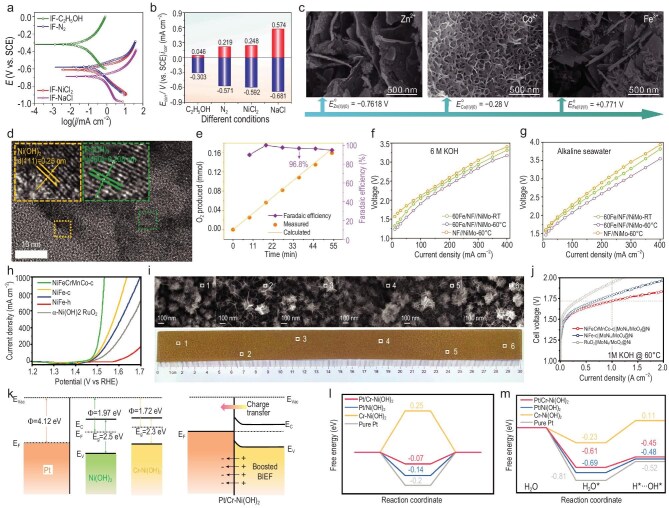
Corrosion engineering of scalable electrocatalysts for high-current-density water electrolysis. (a) Corrosion polarization curves. (b) Corrosion potentials and current densities comparison. (c) SEM images of Zn^2+^, Co^2+^ and Fe^3+^ corrosion. (a–c) Reprinted with permission from Liu *et al.* [90]. Copyright 2021, Royal Society of Chemistry. (d) TEM image of 60Fe/NF. (e) Faradaic efficiency, and theoretical and experimentally measured O_2_ production. Polarization curves in (f) 6.0 M KOH and (g) alkaline seawater. (d–g) Reprinted with permission from Zhuo *et al.* [115]. Copyright 2023, Wiley‐VCH. (h) LSV curves in 1.0 M KOH. (i) Optical graph and SEM images of large-sized NiFeCrMnCo-c. (j) LSV curves for AEMWE in 1.0 M KOH at 60°C. (h–j) Reprinted with permission from Yuan *et al.* [116]. Copyright 2025, Wiley‐VCH. (k) Scheme for electronic structure modification. (l) Gibbs free energy of hydrogen intermediate. (m) Transition-state energy barrier diagram of H_2_O dissociation. (k–m) Reprinted with permission from Yuan *et al.* [117] Copyright 2025, American Chemical Society. E, potential; RHE, reversible hydrogen electrode; SCE, saturated calomel electrode; ϕ, work functions.

Since the corrosion process is subject to various factors, Wang’s group selected chloride solutions to corrode IF to investigate the corrosion mechanisms and fabricate efficient OER electrocatalysts [90]. By analyzing photographs, SEM images and corrosion polarization curves, the corrosion behaviors were investigated by logically replacing Ni^2+^, H_2_O and O_2_ with Na^+^, C_2_H_5_OH and N_2_, and it was found that Ni^2+^, H_2_O and O_2_ were indispensable in corrosion engineering for generating uniform corrosion layers on IF (IF-NiCl_2_), which might further affect the electrocatalytic performance. Moreover, the corrosion potentials and current densities were increased in the order of IF-C_2_H_5_OH, IF-N_2_, IF-NiCl_2_ and IF-NaCl, indicating the increased corrosion degree (Fig. 6a and b). By further comparison, all of H_2_O, O_2_ and Ni^2+^ are essential for fabricating uniform IF-NiCl_2_. The corrosion behaviors in Zn^2+^, Co^2+^ and Fe^3+^ chloride solutions were then probed. The microstructures of the corrosion layers with CoCl_2_ were found to be similar to those in NiCl_2_, suggesting that suitable electrode potentials were also responsible for the formation of uniform corrosion layers (Fig. 6c). As a result, IF-NiCl_2_ offered a low overpotential of 291 mV and high long-term stability, with 100 h at 500 mA cm^−2^ in 1.0 M KOH. Meanwhile, a large IF-NiCl_2_ (8 cm × 8 cm) was also prepared with uniform corrosion layers, showing unobservable performance degradation. When further doping Ru^3+^ into IF-NiCl_2_ (IF-NiCl_2_/RuCl_3_) to couple with IF-NiCl_2_, a small cell voltage of 1.81 V was obtained at 500 mA cm^−2^ in 1.0 M KOH. Through immersing NF in Fe(NO_3_)_3_ solutions, Ni–Fe hydroxide-based OER electrocatalysts were synthesized [115]. Through the variation of the immersion time and Fe(NO_3_)_3_ concentrations, 60 s and 0.2 M Fe(NO_3_)_3_ were found to produce 60Fe/NF, composed of Ni(OH)_2_ and Fe(OH)_3_ (Fig. 6d), exhibiting optimal OER performance. It delivered 240 and 680 mV at 100 and 1000 mA cm^−2^, respectively. Excellent durability and high Faradaic efficiency were also obtained (Fig. 6e). Furthermore, 60Fe/NF demonstrated excellent performance in alkaline seawater. HRTEM and *in situ* Raman analysis revealed the transformation of 60Fe/NF into Ni(Fe)OOH, and proved that the Fe decoration contributed to promotion of OER. Moreover, 60Fe/NF could be easily scaled up to 2000 cm^2^ (10 cm × 2 m) without apparent loss of electrocatalytic activity. For further water electrolysis applications, 60Fe/NF was coupled with NiMo, and the pair showed 3.4 and 3.9 V at 400 mA cm^−2^ in 6.0 M KOH and alkaline seawater (60°C), respectively (Fig. 6f and g), as well as high durability at 100 mA cm^−2^ in 6.0 M KOH (60°C). Similarly, Yuan *et al.* fabricated a multi-element low-crystal transition metal hydroxide (NiFeCrMnCo-c) via a one-step corrosion process [116]. Theoretical calculation demonstrated the improved conductivity and reduced energy barrier of NiFeCrMnCo-c owing to the multi-element doping. In light of this, it realized a low overpotential of 303 mV and stability for 300 h at 1000 mA cm^−2^ in 1.0 M KOH. In addition, owing to the rapid and simple synthetic procedure, NiFeCrMnCo-c was prepared on a large scale (30 cm × 2.5 cm) with uniform surface morphology. Furthermore, the NiFeCrMnCo-c-based AEM electrolyzer showed a cell voltage of 1.72 V at 1000 mA cm^−2^ and stable performance for 100 h under 1.0 M KOH and 60°C. Subsequently, Yuan *et al.* also developed a Cr-doped, Pt-loaded nickel hydroxide [Pt/Cr–Ni(OH)_2_] HER electrocatalyst with a large area of ∼75 cm^2^ [117]. SEM and X-ray diffraction (XRD) characterizations of random areas confirm the homogeneity of the large-sized Pt/Cr–Ni(OH)_2_. Further experimental results demonstrated that the work function (*W*_f_) of hydroxides was increased owing to Cr doping, resulting in strengthened built-in electric field (BIEF) and more gathered electrons around Pt, thereby fine-tuning the electron structure of Pt/Cr–Ni(OH)_2_. As a result, Pt/Cr–Ni(OH)_2_ demonstrated small overpotentials of 156 mV at 1000 mA cm^−2^ in 1.0 M KOH. It also exhibited high durability at 50 mA cm^−2^ for 100 h. Moreover, when Pt/Cr–Ni(OH)_2_ was further assembled with NiFe-LDH for AEMWE, the couple delivered 1.75 V at 1000 mA cm^−2^ under 1.0 M KOH and 60°C, which also operated stably at 1000 mA cm^−2^ for 100 h. As further verified by DFT calculations, benefiting from the Cr doping, the water dissociation kinetics were accelerated and the intermediate adsorption energetics were also optimized, thus boosting the HER performance of Pt/Cr–Ni(OH)_2_.

**Figure 7.**
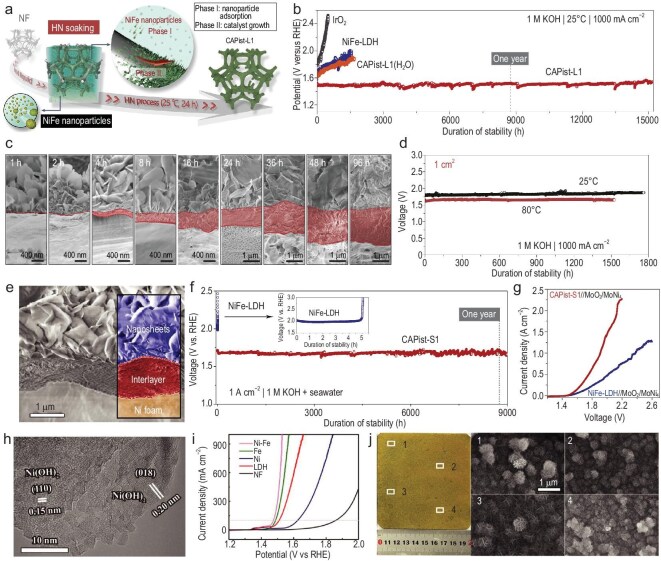
Corrosion engineering of scalable electrocatalysts for high-current-density water electrolysis. (a) Scheme for preparing CAPist-L1. (b) Long-term durability. (c) Cross-sectional SEM images with different soaking time. (d) Stability of the 1 cm^2^ electrolyzer. (a–d) Reprinted with permission from Li *et al.* [118]. Copyright 2024, Springer Nature. (e) Cross-sectional SEM image of CAPist-S1. (f) Durability of CAPist-S1 and NiFe LDH in alkaline natural seawater at 1000 mA cm^−2^. Inset: stability of NiFe LDH. (g) LSV curves of CAPist-S1//MoO_2_/MoNi_4_ and NiFe LDH//MoO_2_/MoNi_4_ at room temperature in alkaline natural seawater. (e–g) Reprinted with permission from Du *et al.* [119]. Copyright 2025, Wiley‐VCH. (h) HRTEM image of Ni-Fe. (i) OER LSV curves. (j) Photograph of NiFe (10 cm × 10 cm) and the corresponding SEM images of 1–4 (white boxes), respectively. (h–j) Reprinted with permission from Du *et al.* [120]. Copyright 2023, Wiley‐VCH. HN, heterogeneous nucleation.

As a recent key breakthrough, Sun’s group developed a NiFe-based electrocatalyst (CAPist-L1) via a seed-assisted heterogeneous nucleation process, achieving ultra-high activity and exceptional long-term durability (Fig. 7a) [106]. The heterogeneous nucleation was realized based on the limited solubility of metal sulfates in organic solvents, leading to the generation of insoluble nanoparticles, and the proposed synthetic process enabled the successful fabrication of 400 cm^2^ (20 cm × 20 cm) CAPist-L1 on both NF and nickel mesh. In 1.0 M KOH, CAPist-L1 presented ultra-high electrocatalytic activity (283 ± 12.7 mV @ 5000 mA cm^−2^) and extraordinary stability (1000 mA cm^−2^ @ 15 200 h) (Fig. 7b). Linear sweep voltammetry (LSV) curves collected from random regions verify its high repeatability and uniformity. Moreover, SEM images revealed that a dense interlayer was presented between the CAPist-L1 electrocatalytic layer and the NF surface, enabling fast electron transfer and offering reinforced structural and mechanical stability in the OER (Fig. 7c). The further AEMWE applications verified that the CAPist-L1**||**Ni_4_Mo/MoO_2_ couple (1 cm^2^ active area) delivered 2.0 V at 7350 mA cm^−2^ with a high Faradaic efficiency (99.29% ± 0.59%). The pair also demonstrated excellent durability at 1000 mA cm^−2^ (1.0 M KOH, 1700 h @ 25°C and 1500 h @ 80°C) and at 2000 mA cm^−2^ (1.0 M KOH, 1200 h @ 25°C) (Fig. 7d). Furthermore, the enlarged pair with 25 cm^2^ active area achieved 1.80 V at 2730 mA cm^−2^ (1.0 M KOH, 60°C) with stable operation at 25 000 mA for 1500 h (1.0 M KOH, 25°C). Subsequently, Sun’s group further adopted the synthetic strategy to fabricate a corrosion-resistant NiFe LDH (CAPist-S1) on NF for seawater oxidation [119]. Similarly, CAPist-S1 was also synthesized on a large scale with an area of 400 cm^2^, consisting of a 1–2 μm dense interlayer between NiFe LDH and NF (Fig. 7e). In alkaline simulated seawater (1.0 M KOH + 0.5 M NaCl) and alkaline natural seawater (1.0 M KOH + seawater), CAPist-S1 delivered 1000 A cm^−2^ at only 200 and 220 mV, respectively, surpassing the conventional hydrothermally synthesized NiFe LDH. Also, benefiting from the generated dense interlayer, CAPist-S1 exhibited enhanced Cl^−^ corrosion resistance, making it stable in alkaline natural seawater at 1000 A cm^−2^ over 9000 h (Fig. 7f). Furthermore, when CAPist-S1 was coupled with a cathodic MoO_2_/MoNi_4_ for alkaline seawater AEM electrolysis, a small cell voltage of 1.91 V was achieved at 1000 A cm^−2^ under room temperature with an outstanding stability over 700 h (Fig. 7g), validating its promising application prospects.

Different from the immersion approach mentioned above, Li’s group reported a scalable coating approach for preparing NiFe (oxy)hydroxide with abundant grain boundaries on NF (NiFe) [120]. HRTEM confirmed the presence of numerous grain boundaries between the crystalline nanograins (Fig. 7e), which originated from the suitable and weak acidic environment provided by Ni^2+^, aiding in providing abundant active sites. Consequently, NiFe was endowed with outstanding activity and durability (Fig. 7f). Also, its electrocatalytic efficiency was as high as >99%. Furthermore, NiFe was scalably synthesized (10 cm × 10 cm) with uniform nanosheet-assembled flowers (Fig. 7g), which also demonstrated high electrocatalytic activity (250 mV @ 1000 mA) and stability (1000 mA @ 100 h).

Overall, considering the high-performance electrocatalysts developed, together with the ease and convenience of corrosion engineering, this strategy underscores the significance of advancing scalable water electrolysis applications.

### Joule heating and combustion

**Figure 8.**
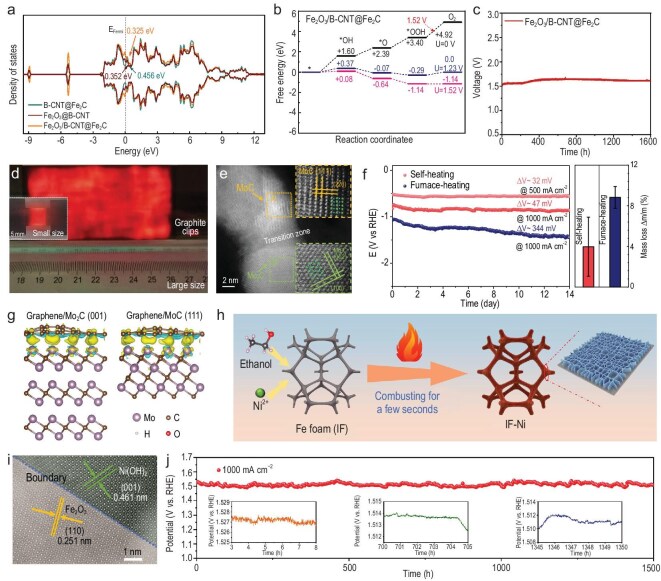
Joule heating and combustion of scalable electrocatalysts for high-current-density water electrolysis. (a) Density of states (DOS) calculations. (b) Gibbs free energy diagrams. (c) Chronopotentiometry testing at 1.0 A cm^−2^. (a–c) Reprinted with permission from Chen *et al.* [121]. Copyright 2024, Wiley‐VCH. (d) Optical images of CNT at high temperature with sizes of 8 cm × 4 cm and 1.5 cm × 0.8 cm (inset). (e) HAADF-STEM image. Inset: HAADF-STEM images and FT patterns. (f) Long-term stability testing and mass loss. (g) Charge density difference. (d–g) Reprinted with permission from Li *et al.* [122] Copyright 2022, Springer Nature. (h) Illustration of IF-Ni preparation. (i) HRTEM image of IF-Ni. (j) Stability of IF-Ni at 1000 mA cm^–2^. (h–j) Reprinted with permission from Yu *et al.* [47] Copyright 2023, American Chemical Society.

In addition to the three aforementioned well-established approaches, other attractive and straightforward synthetic methods have also been reported for the direct and scalable synthesis of high-performance electrocatalysts. Taking Joule heating as an example, owing to the characteristics of instant temperature elevation, swift quenching and periodic pulsing, it has gradually been recognized as a promising way for synthesizing electrocatalysts within an ultra-short period of time. For instance, regarding powder electrocatalyst synthesis, bamboo-like carbon nanotube (B-CNT)-encapsulated Fe_2_C nanoparticle-supported Fe_2_O_3_ nanoclusters (Fe_2_O_3_/B-CNT@Fe_2_C) were fabricated through rapid Joule heating by Chen and co-workers [121]. The rapid synthesis enabled the gram-scale preparation of Fe_2_O_3_/B-CNT@Fe_2_C. Electrocatalytic performance assessment in 1.0 M KOH showed that Fe_2_O_3_/B-CNT@Fe_2_C had a high activity (342 mV @ 100 mA cm^−2^) and high turnover frequency (TOF, 0.73 s^−1^). It also had outstanding stability at 100 mA cm^−2^ for 1200 h. It is revealed that the electronic structure of Fe_2_O_3_ was stabilized and fine-tuned by the B-CNT-encapsulated Fe_2_C through d-p-d orbital coupling (Fig. 8a), thereby realizing moderate adsorption of intermediates and a reduced energy barrier of OOH* (Fig. 8b), thus promoting the OER. When Fe_2_O_3_/B-CNT@Fe_2_C was further assembled with Pt/C for AEMWE, a low cell voltage of 1.48 V and high efficiency of 83.1% were realized at 1000 mA cm^−2^ in 1.0 M KOH at 50°C. The assembled electrolyzer also demonstrated excellent durability for 1600 h (Fig. 8c). In terms of self-supported electrocatalysts, Mo_2_C/MoC/CNT was synthesized by Li and co-workers [122]. Such a rapid preparation approach facilitated the large-scale fabrication of Mo_2_C/MoC/CNT (8 cm × 4 cm) (Fig. 8d). SEM observation confirmed the uniform distribution of Mo_2_C/MoC (∼20 nm) without visible agglomeration, while HRTEM verified the existence of heterointerfaces between Mo_2_C and MoC (Fig. 8e), providing abundant electrocatalytic active sites for the HER. Thus, Mo_2_C/MoC/CNT realized small overpotentials and stable operation of 14 days at 1000 mA cm^−2^, showing a slight increase of overpotential, only 47 mV, in 1.0 M KOH (Fig. 8f). Further DFT calculations demonstrated that the high performance of Mo_2_C/MoC/CNT stemmed from the optimal hydrogen adsorption free energy and strong chemical bonding between Mo_2_C/MoC and CNT (Fig. 8g). Moreover, the presented synthetic approach is universal and enabled the synthesis of well-dispersed single-phase Nb_4_C_3_ and W_2_C/WC. Overall, Joule heating not only reduces the synthetic time but also enables high electrocatalytic efficiency in water electrolysis, bestowing a novel approach for electrocatalyst preparation.

**Figure 9.**
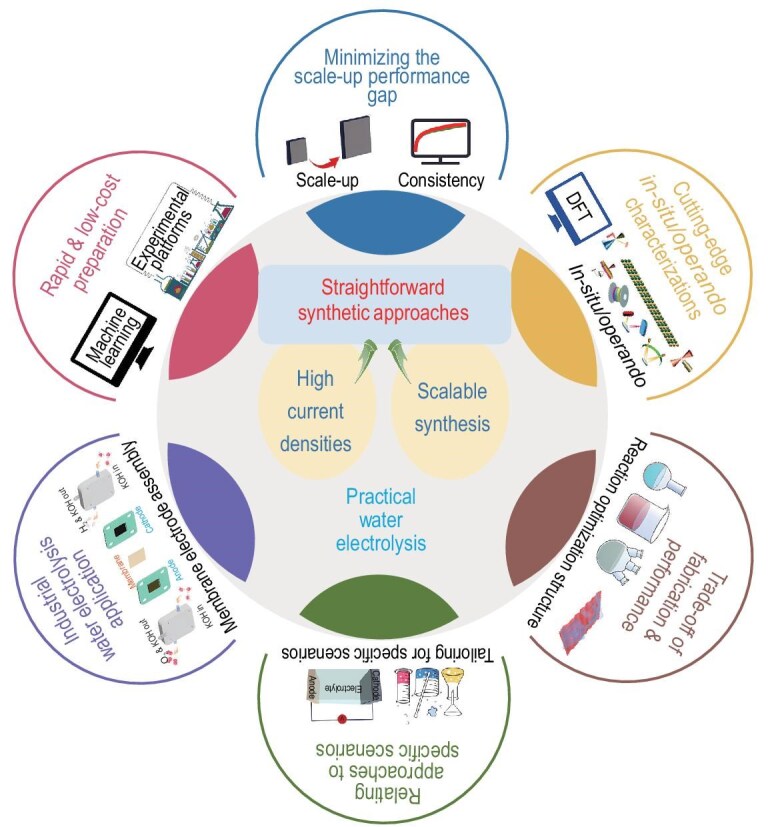
Perspectives of straightforward synthetic approaches for synthesizing large-sized high-performance electrocatalysts for high-current-density water electrolysis.

To achieve high-throughput and scalable electrocatalyst preparation without relying on supplementary equipment, a one-step combustion approach for depositing a series of oxide hybrids on IF within seconds was proposed by Peng’s group [47]. Taking Ni as an example, IF was initially immersed in an ethanol-based NiCl_2_ solution, and then extracted and directly combusted in an atmospheric environment for *in situ* generation of dense Ni(OH)_2_/Fe_2_O_3_ layers (IF-Ni) (Fig. 8h). SEM imaging confirmed that dense and uniform nanosheets were distributed on IF, while HRTEM and high-angle annular dark-field scanning transmission electron microscopy (HAADF-STEM) imaging demonstrated the existence of Ni(OH)_2_ and Fe_2_O_3_, and the boundary between Ni(OH)_2_ and Fe_2_O_3_ (Fig. 8i). Due to the operational simplicity, a large-sized preparation of 400 cm^2^ IF-Ni (20 cm × 20 cm) was realized. Moreover, this proposed approach was also universal, enabling the fast synthesis of IF-Mn, IF-Fe, IF-Co and IF-Zn using the corresponding ethanol solutions of metal chlorides. For OER, IF-Ni exhibited outstanding electrocatalytic activity and fast kinetics in 1.0 M KOH, achieving 271 mV at 1000 mA cm^–2^ and 52.83 mV dec^–1^. It also demonstrated a nearly 100% Faradaic efficiency and superb durability at 1000 mA cm^−2^ for 1500 h (Fig. 8j). The further coupling of IF-Ni and MoNi_4_ for water electrolysis achieved 1.72 V at 1000 mA cm^–2^. Moreover, DFT calculations revealed that the electron distribution was modulated by the constructive synergy, which enhanced adsorption activity of Fe and accelerated OER kinetics. This example underscores the significant potential of combustion for scalable electrocatalyst production in industrial applications.

## SUMMARY AND PROSPECTS

In this review, recent advancements in straightforward synthetic approaches for scalable electrocatalyst preparation for water electrolysis are comprehensively summarized. The highlighted approaches, including hydrothermal and solvothermal synthesis, electrodeposition, corrosion engineering, Joule heating and combustion, are introduced in detail owing to their operational simplicity, effective modification, universality and scalability. To present the advancements, a comprehensive evaluation encompassing critical electrocatalytic performance metrics, scaled-up sizes and water electrolysis applications are outlined in Tables 2 and 3. Current studies have demonstrated that the aforementioned straightforward synthetic approaches are both significant and effective, whether in fundamental research or practical applications. Notwithstanding the substantial achievements, key challenges persist and need to be overcome in future investigations (Fig. 9).

**Table 2.** Summary of the properties including synthesized sizes or weights, electrocatalytic activity and durability of electrocatalysts prepared through straightforward synthetic approaches.

Straightforward synthetic approach	Electrocatalyst	Electrode size (cm^2^)	Electrolyte	OER/HER	Overpotential^a^ (mV @ mA cm^−2^)	Stability^a^ (mA cm^−2^ @ h)	Ref.
Hydrothermal and solvothermal synthesis	Co, Mo-NiFe LDH	∼19.5	1.0 M KOH	OER	272 @ 200	100 @ 100	[98]
	Fe-NiOOH/CQDs	^b^	1.0 M KOH	OER	450 @ 1000	10 @ 80	[99]
	FeNiHOF	90	1.0 M KOH	OER	340 @ 2000	1000 @ 1000	[100]
	NiFe-BTC-GNPs MOF	^b^	1.0 M KOH	OER	220 @ 10 (on carbon-fiber paper)	≥ 1000^c^	[101]
					180 @ 10 (on NF)		
Electrodeposition	NNAs	2000	1.0 M KOH	HER	469 @ 5000	1000 @ 7000	[103]
	ND-Ni	∼20	1.0 M KOH	HER	230 @ 500	500 @ 150	[104]
	FeCoNiMnOOH/NF	≥100	1.0 M KOH	OER	441 @ 1000	100 @ 200	[108]
						500 @ 200	
			6.0 M KOH			500 @ 100 (60°C)	
	CoFeWO_x_	60	1.0 M KOH	OER	280 @ 500		[109]
	Zn-(Ni/FeOOH)@NF	40	1.0 M KOH	OER	347 @ 1500	1000 @ 1000	[110]
Corrosion engineering	O_2_-Cat-1	1000	1.0 M KOH	OER	269 @ 10	100 @ 100	[83]
	3D-O_2_-Cat-1			OER	340 @ 1000	1000 @ 5000	
			10.0 M KOH	OER	280 @ 1000	1000 @ 1050	
	(Ni, Fe)_3_S_2_/NFF	5000	30% KOH	OER	241 @ 10^d^	100 @ 1000	[114]
				HER	168 @ 100^d^	100 @ 1000	
	Ni(Fe)OOH/Ni(Fe)S_x_	100	1.0 M KOH	OER	313 @ 1000	100 @ 100	[84]
	IF-NiCl_2_	64	1.0 M KOH	OER	291 @ 500	500 @ 100	[90]
	60Fe/NF	2000	1.0 M KOH	OER	680 @ 1000	250 @ 100	[115]
	NiFeCrMnCo-c	75	1.0 M KOH	OER	303 @ 1000	1000 @ 300	[116]
	Pt/Cr–Ni(OH)_2_	∼75	1.0 M KOH	HER	156 @ 1000	50 @ 100	[117]
	CAPist-L1	400	1.0 M KOH	OER	283 ± 12.7 @ 5000	1000 @ 15 200	[118]
	CAPist-S1	400	1.0 M KOH + 0.5 M NaCl	OER	200 @ 1000		[119]
			1.0 M KOH + seawater		220 @ 1000	1000 @ 9000	
	Ni-Fe	100	1.0 M KOH	OER	296 @ 1000	500 @ 500	[120]
Joule heating	Fe_2_O_3_/B-CNT@Fe_2_C	0.9140 g^e^	1.0 M KOH	OER	342 @ 100	100 @ 1200	[121]
	Mo_2_C/MoC/CNT	32	1.0 M KOH	HER	255 @ 1500	1000 @ 336	[122]
Combustion	IF-Ni	400	1.0 M KOH	OER	271 @ 1000	1000 @ 1500	[47]

a Measured at 25°C, unless explicitly stated. ^b^No clear indication of the scalable extent. ^c^Considering the overall stability in both half-cell and the single-cell studies. ^d^Without *iR* correction. ^e^Weight in grams.

**Table 3.** Water electrolysis performance of the electrocatalysts.

Electrocatalyst couple								
Anode	Cathode	Water electrolysis type	Electrode Size (cm^2^)	Electrolyte	Temperature (°C)	Voltage^a^ (V @ mA cm^−2^)	Stability^a^ (mA cm^−2^ @ h)	Ref.
Co, Mo-NiFe LDH	Pt/C	AEMWE	∼4	1.0 M KOH	25	1.94 @ 500	∼500 @ 130	[98]
Co, Mo-NiFe LDH	Raney Ni	AWE	∼19.5	30% KOH	85	1.91 @ 500	400 @ 100	
Fe-NiOOH/CQDs	Pt/C	AWE	1	1.0 M KOH	25	1.85 @ 100	500 @ 12	[99]
FeNiHOF	Raney Ni	AWE	9	1.0 M KOH	25	1.87 @ 1000		[100]
				6.0 M KOH	25	1.81 @ 1000	1000 @ 500	
NiFe-BTC-GNPs MOF	MoNi_4_/MoO_2_	AEMWE	4	0.1 M KOH	70	1.85 @ 1150		[101]
				DI water	50		460 @ ∼75	
					70	1.85 @ 540	540 @ ∼45	
NiFeOH	ND-Ni	AWE	∼20	1.0 M KOH	25	1.79 @ 1000		[104]
				6.0 M KOH	∼80	1.71 @ 1000	500 @ 300	
							1000 @ 800	
CoFeWO_x_	NiMoO_4_	AWE	1	1.0 M KOH	25		100 @ 120	[109]
				10.0 M KOH	80		500 @ 48	
(Ni, Fe)_3_S_2_/NFF	(Ni, Fe)_3_S_2_/NFF	AWE	4	30% KOH	80	1.93 @ 600	600 @ 420	[114]
			∼314		80	∼1.95 @ 300	300 @ 216	
Ni(Fe)OOH/Ni(Fe)S_x_	Mo_2_C/MoO_2_/MoNi_4_	AWE	1	1.0 M KOH	25	1.809 @ 1000	100 @ 100	[84]
				30% KOH	60	1.605 @ 1000		
					70	1.58 @ 1000		
					90	1.565 @ 1000		
IF-NiCl_2_	IF-NiCl_2_/RuCl_3_	AWE	1	1.0 M KOH	25	1.81 @ 500	500 @ 48	[90]
60Fe/NF	NiMo	AWE	20.25	6.0 M KOH	60	3.4 @ 400	100 @ 20	[115]
NiFeCrMnCo-c	MoNi_4_/MoO_2_	AEMWE	1	1.0 M KOH	60	1.72 @ 1000	1000 @ 100	[116]
NiFe LDH	Pt/Cr–Ni(OH)_2_	AEMWE	1	1.0 M KOH	60	1.75 @ 1000	1000 @ 100	[117]
CAPist-L1	Ni_4_Mo/MoO_2_	AEMWE	1	1.0 M KOH	25		1000 @ 1700	[118]
					25		2000 @ 1200	
					80	2.0 @ 7350	1000 @ 1500	
			25		60	1.8 @ 2730	1000 @ 1500	
CAPist-S1	Ni_4_Mo/MoO_2_	AEMWE	1	1.0 M KOH + seawater	25	1.91 @ 1000	1000 @ 700	[119]
Fe_2_O_3_/B-CNT@Fe_2_C	Pt/C	AEMWE	1	1.0 M KOH	50	1.48 @ 1000	1000 @ 1600	[121]
IF-Ni	MoNi_4_	AWE	1 cm^2^	1.0 M KOH	25	1.72 @ 1000		[47]

a Measured at 25°C, unless explicitly stated.

### Rapid and low-cost preparation

Despite the widespread application of cost-effective and robust nickel-based catalysts in industrial water electrolysis, several directions are still needed to be further advanced to promote practical water electrolysis applications, among which developing high-performance electrocatalysts with both rapid large-scale production capabilities and low manufacturing costs are the prerequisites to transfer from the laboratory to industry. In this regard, efforts can be made in the following aspects. Integrating machine learning-based computational screening with high-throughput experimental platforms contributes to systematic development of highly efficient electrocatalysts [123]. Meanwhile, the utilization of non-precious or low-precious metals enables the realization of low-cost electrocatalyst preparation. Also, recovering precious metals from spent electrodes or regenerating deactivated electrocatalysts contribute to enhancing cost sustainability. Moreover, driving the development of high-throughput preparation technologies (e.g. 3D printing) will contribute to balancing the synthesis rate and productivity of electrocatalysts without compromising the electrocatalytic performance. Furthermore, adopting strategies like alloying, doping and strain/defect engineering, along with the increase of surface roughness, is expected to enhance intrinsic activity and improve surface wettability while ensuring rapid and low-cost fabrication [124].

### Cutting-edge *in situ/operando* characterizations

Advancing the development of characterization techniques [X-ray fine structure (XAFS), X-ray photoelectron spectroscopy (XPS), TEM and Raman spectroscopy) with high temporal and spatial resolution facilitates the real-time tracking of dynamic catalytic processes and atomic-level structural evolution, thereby uncovering the origins of activity. Simultaneously, establishing theoretical models aids experimental verification, thereby enabling synergy with experiments and rational electrocatalyst fabrication. On basis of these, leveraging and upgrading *in situ/operando* characterizations for water electrolyzers under simulated industrial conditions will further provide valuable insights into the reaction process, such as reaction intermediates, real active sites and preferred reaction mechanisms. Furthermore, tailoring characterization techniques for practical water electrolysis devices under actual conditions is expected to gain effective operational information, including morphological evaluation, bubble generation and dissipation, electrocatalyst reconstruction and dissolution, which is beneficial for electrocatalyst optimization and water electrolysis industrialization.

### Trade-off of scalable fabrication and high performance

Based on the straightforward synthetic approaches, realizing high activity and stability of scalable electrocatalysts is conducive to sustainable development. In addition, despite advances in scalable electrocatalyst fabrication (predominantly ≤400 cm^2^), few of them meet the 2050 size targets set by the International Renewable Energy Agency (IRENA) (1000 cm^2^ for AEMWE, and 30 000 cm^2^ for AWE), and still fail to fully satisfy industrial application requirements [125]. In this regard, the following aspects can be fully considered. In the pursuit of straightforward synthesis, the optimization of reaction parameters, including the temperature, time, metal salts and solvents aids regulation of the electrocatalytic performance and scaling up the dimensions or weights. Meanwhile, selecting eco-friendly reagents is equally important to ensure sustainable and environmentally friendly electrocatalyst preparation. During the structural design, designing nanoporous structures facilitates the enhancement of electrocatalytic active surface area and density of active sites, and promotes the diffusion of reactants and products, thus enhancing electrocatalytic activity. Leveraging high-throughput screening enables precise prediction of performance and minimizes trial-and-error costs, thereby preventing research from staying at the lab scale and allowing for the feasibility of industrial-scale application.

### Minimizing the scale-up performance gap

Current studies have verified the feasibility of scalable catalyst preparation. In order to maintain the structure and catalytic performance upon scale-up, the reaction conditions such as the concentration and ratio of reactants, and reaction temperature and time should be precisely controlled. Step-by-step scaling-up is necessary to ensure the reproducibility, which will contribute to the eventual industrial-scale utilization. Generally, the uniformity of the structure and performance can be demonstrated by comparing the results from characterization techniques (e.g. SEM and TEM) and performance testing (e.g. LSV curves) before and after scale-up [115–117]. While it has proved to be a highly effective approach, it should be noted that it is not lossless. The involved analysis process will compromise the structural integrity of the catalyst, especially for self-supporting electrodes, which hinders further scale-up assessment. Therefore, the development and utilization of *in situ*/*ex situ* non-destructive characterizations, such as SEM, TEM, XRD, Raman and Fourier transform infrared spectrometry (FTIR), are of critical importance.

While maintaining the catalyst’s performance before and after scalable preparation, narrowing the performance gap from small-scale testing to large-scale electrolyzers is equally important. Currently, most catalysts demonstrate excellent performance in small-scale tests, but they often suffer significant performance degradation when incorporated into larger-scale electrolyzers [118,126]. To minimize the performance difference, the following aspects should be taken into account: (i) maintaining the consistency in the test environment as much as possible, such as temperature, pressure and electrolyte concentration, which is the most basic requirement; (ii) ensuring effective bubble removal and electrolyte flow. Efficient bubble removal facilitates bubble detachment from the catalyst surface and ensures efficient gas–liquid interface reaction, guaranteeing effective water distribution and transport within the electrolyzer. Therefore, designing hydrophilic and aerophobic electrodes and optimizing the electrolyte flow rate are also necessary; and (iii) optimizing the electrolyzer configurations and components. From small-scale to large-scale electrolyzers, the differences in the mass transfer losses and electrochemical resistances lead to the discrepancy in performance. In this regard, it is beneficial to optimize the components like gasket, end plate and current collector, as well as provide adequate water supply and discharge channels and gas channels, which can compensate for the performance gap. Moreover, the proper integration and assembly of all components cannot be overlooked.

### Industrial water electrolysis application

Under lab-scale conditions, various electrocatalysts have demonstrated outstanding long-term stability under large current densities (e.g. ≥1000 mA cm^−2^) for over 1000 h and in electrolytes with varying concentrations (i.e. 1.0 M, 6.0 M or 30% KOH). Nevertheless, the ultimate goal of developing high-performance electrocatalysts is to apply them in actual water electrolysis systems, but there are few reports available at present, making it difficult to evaluate the potential in practical applications. Therefore, scaling up electrocatalysts from the lab scale to the pilot scale, and eventually to the industrial scale, is of critical importance for industrial-level water electrolysis. Along with scalable fabrication, the electrocatalysts need to ensure stable operation under demanding conditions, such as temperatures ranging from 50°C to 80°C and current densities of ≥1000 mA cm^−2^. In addition, during the assembly of the membrane electrode, the systematic optimization of mass transfer, electrochemical and interfacial resistances, the gas bubble effect and the flow field requires comprehensive consideration. Moreover, for large-scale stacks and low-grade electrolytes (e.g. seawater), one of the main concerns lies in maintaining the high performance of the electrocatalysts. Current research on anion engineering has provided a viable pathway, including the adsorption of Cl^−^ [127], the chemical fixation of free SO_4_^2−^ [128] and anchoring basic anions (i.e. PO_4_^3−^) [129], which is beneficial for suppressing metal leaching, regulating the reaction mechanism, enhancing electrocatalytic activity and improving corrosion resistance. Building upon the progress, developing additional effective modulation strategies and the practical application in real seawater need further investigation. On the other hand, natural seawater with complex components is also a critical issue. The predominant Na^+^ and Cl^−^ will accelerate electrocatalyst corrosion and degradation, while Ca^2+^- and Mg^2+^-induced precipitates, along with impurities and microorganisms, can also poison electrocatalysts and block device components, leading to deterioration of catalytic performance [130–133]. Therefore, it is crucial to develop effective strategies to mitigate the adverse effects of complex composition on seawater electrolysis systems. Currently, the pre-desalination of seawater and the utilization of reverse osmosis (RO) or forward osmosis (FO) technologies are emerging as feasible approaches, yet cost reduction still requires further optimization [134–136]. With respect to AWE and AEMWE, some improvements should also be considered. For AWE, decreasing the thickness of diaphragms can lower the resistance, thereby reducing electricity consumption, achieving high current densities and improving cell efficiency. Meanwhile, thinner diaphragms are able to mitigate gas crossover risks and improve the generated gas purity. Regarding AEMWE, simultaneously enhancing the chemical, mechanical and thermal stability of membranes while developing highly conducting polymer compositions is beneficial for increasing the durability, while advancing the industrialization, conducting techno-economic analysis (TEA) and life-cycle assessment (LCA) are indispensable to assess and validate the viability of green hydrogen production for a low-carbon economy [137,138]. Through TEA and LCA, key parameters that affect the capital expenditures and environmental trade-offs can be systematically analyzed to optimize priorities for realizing energy sustainability.

### Relating synthetic approaches to specific application scenarios

Given the characteristics of the aforementioned various straightforward synthetic approaches, they have all made significant advancements in water electrolysis regarding high current densities and large-scale preparation. Building on this, establishing the relationship between different synthesis approaches and specific application scenarios, such as lab-scale testing, pilot-scale trials and industrial-scale production, is of paramount importance. Specifically, hydro/solvothermal synthesis is the preferred option when precise control of morphology and crystalline phase is required. Taking a typical electrocatalyst NiCo_2_O_4_ as an example, the nanosheet-structured NiCo_2_O_4_ with (100) exposed crystal planes has higher surface energy and lower adsorption free energy of H^∗^ in the HER and smaller theoretical OER overpotential compared to octahedral NiCo_2_O_4_ with (111) exposed crystal planes and truncated octahedral NiCo_2_O_4_ with (111) and (100) exposed crystal planes. This result validates the critical role of precise crystal plane and morphology control in optimizing electrocatalytic performance, which is beneficial for guiding catalyst application in pilot-scale trials and industrial-scale production [139]. However, owing to the pressure equipment costs and continuous production issues, its scalability is restricted and it is currently applicable from lab-scale to pilot-scale application scenarios. Regarding electrodeposition, corrosion engineering, Joule heating and combustion, they feature relatively shorter synthesis times and ease of scale-up. Nevertheless, due to the challenges in the structural and morphological control for corrosion engineering and combustion, and the limited reactor sizes for Joule heating, they are currently insufficient for industrial-level application scenarios and remain primarily at the laboratory stage. However, for electrodeposition, despite the potential issues of irregular deposition and non-uniform thickness, the developed technologies (e.g. roll-to-roll) enable fast, scalable, low-cost and continuous electrocatalyst production, making it more viable for industrial application scenarios [140]. While achieving these achievements, mitigating or resolving the uniformity of large-sized electrocatalysts and their mechanical stability under industrial current densities remain as challenges, requiring further efforts.

In conclusion, the advancements of the straightforward synthetic approaches for scalable construction of high-performance electrocatalysts for water electrolysis under high current densities have been thoroughly discussed. The preliminary investigations demonstrate that novel electrocatalysts can be prepared on a large scale without the need for complicated and tedious procedures. Not only limited to water electrolysis, these electrocatalysts might also be applicable in other electrocatalysis-related fields, such as electrooxidation of organics, oxidation of hydrazine and catalytic conversion of CO_2_, thus broadening the domains of application of these straightforward synthetic approaches and facilitating their sustainable development.
